# Structural insights into *Escherichia coli* CsgA amyloid fibril assembly revisited

**DOI:** 10.1128/mbio.00795-25

**Published:** 2025-07-11

**Authors:** Mike Sleutel, Han Remaut

**Affiliations:** 1 Structural Biology Brussels, Vrije Universiteit Brussel, Brussels, Belgium; 2 Structural and Molecular Microbiology, VIB-VUB Center for Structural Biology, Brussels, Belgium; University of Michigan-Ann Arbor, Ann Arbor, Michigan, USA

## Abstract

Curli are a widespread component of proteobacterial biofilms. They are functional amyloid fibers that aid in structuring the extracellular matrix that surrounds these multicellular communities. As a functional amyloid, they are the product of a dedicated assembly pathway that needs to navigate the cytotoxicity classically associated with most amyloid depositions. Recent breakthroughs in cryo-EM and protein structure prediction have started to shed light on the unique structural principles of curli fibrils. Here, we revisit the recent literature and focus on the consensus model for these remarkable protein fibrils.

**IMPORTANCE:**

Amyloid fibrils receive enormous interest as instigators of protein aggregation disorders and associated wasting diseases, as functional components of complex biological matrices, including biofilms, and/or as versatile and self-assembling protein scaffolds for protein material applications. Central to each of these phenomena are the structural properties of the component subunits and how they assemble in the amyloid fibril. A thorough understanding of these architectural principles is essential for the rational interference with amyloids, be it inhibitory in the case of disease-associated amyloids, or as a basis for biotechnological applications of functional amyloids.

## MATTERS ARISING

Thirty-six years have passed since the original discovery by Olsén, Jonsson, and Normark (1) of a novel type of *Escherichia coli* surface organelles that facilitate and promote biofilm formation on a host of (a)biotic surfaces. These novel extracellular features of *E. coli* were shown to be “coiled surface structures composed of a single type of subunit, the curlin.” The term “curli” was given due to the typical curvature that these fibers exhibit, and their curling nature has become one of the defining traits of curli. Another curli hallmark only became apparent in 2002 when Chapman et al*.* (2) showed that curli are a bacterial amyloid. An unexpected result at the time, it fully upended the prevailing notion that the “amyloid fold” (if such a thing exists) was uniquely associated with extracellular deposits and plaques isolated from patients suffering from (mostly neurological) wasting disorders. Curli defined a new superfamily of proteins that have evolved to adopt an “amyloid-like” fold characterized by cross-beta stacking, nucleation-dependent polymerization kinetics, and classic amyloid tinctorial properties (3). To reflect the fact that these proteins fulfil specific, seemingly purpose-built roles in biology, we have come to refer to them as “functional amyloids.” Progress on the structural characterization of curli fibers has been slow, but consistent and consecutive breakthroughs slowly brought the general curlin fold into sharper focus. In 2009, solid-state nuclear magnetic resonance revealed a beta-solenoidal architecture (4), which was later supported in 2014 by an atomic curlin monomer model derived from intramolecular co-evolutionary coupling analysis (5). The handedness of the β-helix and the mode of inter-curlin coupling remained unclear at the time.

In 2023, we proposed a model for the curli fiber ultrastructure based on AlphaFold2-multimer modeling and cryo-EM data (6). Importantly, due to the propensity of recombinant *E. coli* CsgA (the major curlin subunit) to laterally self-assemble into larger super bundles of variable diameter, we focused on a CsgA homolog—dubbed R15.5 owing to its number of curli repeats—from *Pontibacter korlensis*, a gram-negative bacterium isolated from the desert of Xinjiang, China. R15.5 was selected because its (predicted) net negative surface charge (pI = 2.98) makes it less prone to aggregation, and thus more amenable to helical reconstruction via cryo-EM. Crucially, our final cryo-EM volume was still suffering from a number of artefacts due to (i) the missing wedge problem as a result of strong preferential orientation and (ii) the inability of image alignment algorithms to find the correct register for the R15.5 protomer, as well as the fibril polarity—a direct consequence of the symmetrical nature of curlins and the absence of low-resolution features to guide particle alignment. Taking into account these limitations, we employed disulfide crosslinking to distinguish between the possible intermolecular interfaces, i.e., consecutive head-to-tail (N/C) interfaces, or alternating head-to-head (N/N) and tail-to-tail (C/C) interactions. From those two options, only the former (N/C stacking) constitutes a polar fiber, whereas the latter is apolar and would therefore have symmetric elongation kinetics, i.e., both poles would extend at similar rates. *In situ* AFM measurements showed asymmetric extension rates in agreement with a polar architecture also suggested by the disulfide cross-linking (6). Earlier work by us showed that recombinant *E. coli* curli fibers have a fast and slow growing pole—favoring a polar curli architecture for *E. coli* as well (7). The R15.5 model that we published consisted of a single, polar protofilament with a 2_1_ screw axis, where R15.5 curlin subunits interact in a head-to-tail manner (i.e., the C-terminal repeat of subunit *i* docks onto the first N-terminal repeat of subunit *i* + 1).

Recently, Bu et al*.* (8) published an atomic model for the protofilament architecture of *E. coli* curli. The authors worked with an engineered CsgA sequence (A63C and V140C) developed by Balistreri et al. (9) due to its increased expression yield and triggerable polymerization through the introduction of a reducing agent. Because these residues do not map onto gatekeeper positions (10) and there is no discernible impact on fibril morphology with respect to wild-type fibers (9), Bu et al*.* (8) assert that these mutations do not affect the CsgA fibril morphology. The authors presented a 218 × 218 pixel cryo-EM reconstruction of CsgA A63C V140C (covering approximately eight protomers), rendered at 3.62 Å resolution. The authors point out that the map quality is limited as a result of preferential orientation artefacts and estimate the “real resolution” to be closer to “4–6 Å.” Despite obvious map limitations, Bu et al*.* present a detailed model (8ENQ) for the protofilament with a remarkably specific succession of curlin protomers in various, alternating orientations: A-A-B-A-A-B-B, where A and B correspond to C→N and N→C protomer orientations along the fiber axis, respectively. The reporting and depositing of this asymmetric and specific architecture is puzzling because the authors stated that—as a result of map quality limitations—“unambiguous determination of monomer connectivity was not possible*”* and “the detailed interactions between these molecules within a fibril need to be revealed by further higher-resolution structures.” Moreover, the proposed *E. coli* curli model would (in all likelihood) be apolar, which is in disagreement with the aforementioned asymmetric elongation kinetics (7). Second, this non-homogeneous succession of subunit orientations is inconsistent with the homogeneous nanogold-NTA labeling of Chen et al. (11) on CsgA_His curli. Finally, it is difficult to see how a random distribution of A and B protomers would yield a consistent curvature of fixed sign (7), taking the proverbial “curl” out of “curli.” We conclude that the cryo-EM maps do not allow for unambiguous assignment of the orientation of individual subunits and that the proposed model is not supported by the experimental data. Therefore, it did not warrant deposition into the Protein Data Bank.

Curli fibers have been historically difficult to characterize structurally, and a sustained future effort is required to fill in the missing details. With the unsubstantiated claims on the subunit connectivity in the *E. coli* CsgA fiber structure, the contribution of Bu et al*.* (8) has clouded our current understanding of curli architecture and risks misguiding structure-based experimental designs or curli-based materials. By now, it is well documented that cryo-EM reconstruction of protein filaments can lead to ambiguous results (12). One way of dealing with such uncertainties is by uploading raw, non-motion-corrected movies to the Electron Microscopy Public Image Archive database (13). This allows for independent, third-party analysis of the cryo-EM data—a strategy that has been fruitful for data sets that have led to conflicting interpretations in the past (14).
